# Head‐to‐head comparison of the diagnostic value of fecal and serum carcinoembryonic antigen for colorectal cancer detection

**DOI:** 10.1002/ijc.70435

**Published:** 2026-04-09

**Authors:** Xianzhe Li, Zitong Zhao, Lara Stassen, Anjana Pradeep Maya, Megha Bhardwaj, Teresa Seum, Janhavi R. Raut, Tafirenyika Gwenzi, Michael Hoffmeister, Petra Schrotz‐King, Hermann Brenner

**Affiliations:** ^1^ Division of Clinical Epidemiology of Early Cancer Detection German Cancer Research Center (DKFZ) Heidelberg Germany; ^2^ Medical Faculty Heidelberg Heidelberg University Heidelberg Germany; ^3^ National Center for Tumor Diseases (NCT) NCT Heidelberg, a Partnership Between DKFZ and University Hospital Heidelberg Heidelberg Germany; ^4^ Division of Primary Cancer Prevention German Cancer Research Center (DKFZ) Heidelberg Germany; ^5^ Cancer Prevention Graduate School German Cancer Research Center (DKFZ) Heidelberg Germany

**Keywords:** biomarker, carcinoembryonic antigen, colorectal cancer, detection

## Abstract

This study aimed to conduct a head‐to‐head comparison of the diagnostic value of serum carcinoembryonic antigen (sCEA) and fecal CEA (fCEA) for colorectal cancer (CRC) detection. Fecal and serum samples from 80 CRC cases at various tumor stages and 100 controls free of colorectal neoplasms at screening colonoscopy were randomly selected from two ongoing large prospective CRC detection studies (IDA and BLITZ) for CEA measurements. Fecal samples were processed using two methods: with and without mechanical homogenization. Diagnostic performance (area under the curve value [AUC], sensitivity) of fCEA and sCEA was compared individually and in combination with fecal immunochemical test (FIT). The fCEA concentrations obtained using both sample processing methods were highly correlated in both CRC cases and controls, but neither correlated with sCEA. The sCEA concentrations demonstrated significantly greater differences between the CRC and control group compared to fCEA concentrations. The diagnostic performance of fCEA obtained with both fecal sample processing methods was significantly lower than that of sCEA (AUC: 0.62 and 0.57 vs. 0.83, both *p* < .001; sensitivity at 85% specificity: 36.2% and 26.2% vs. 52.5%, *p* = .067 and .002). Algorithms combining sCEA with fCEA did not significantly improve the diagnostic performance compared to sCEA alone. Combining FIT with sCEA improved diagnostic performance over FIT alone. However, combining FIT with fCEA showed no improvement. In conclusion, fCEA is inferior to sCEA as a non‐invasive biomarker for CRC detection. Combination of FIT with sCEA demonstrates greater potential for CRC screening than combination of FIT with fCEA.

## Graphical abstract

What's new?

The diagnostic value of fecal versus serum carcinoembryonic antigen (CEA) for colorectal cancer detection remains unclear. This study provides a head‐to‐head comparison of serum and fecal CEA using matched samples from two large colorectal cancer screening studies and two fecal sample processing methods. The results suggest that fecal CEA is markedly inferior to serum CEA in diagnostic performance. Combining serum CEA assessment with fecal immunochemical testing (FIT) improved diagnostic performance over FIT alone. These findings highlight the limited utility of fecal CEA and support prioritizing serum CEA, particularly in combination with FIT, for colorectal cancer detection.

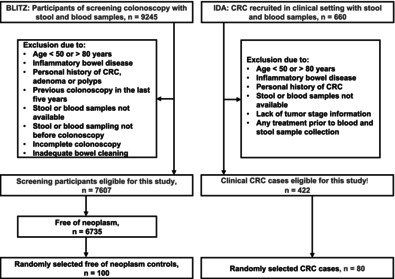

**Abbreviations**
AUC: area under the curve
BLITZ: Begleitende Evaluierung innovativer Testverfahren zur Darmkrebsfrüherkennung (German name)
BMI: body mass index
CI: confidence interval
CRC: colorectal cancer
DKFZ: German Cancer Research Center
fCEA: fecal carcinoembryonic antigen
FIT: fecal immunochemical test
FOBT: fecal occult blood testing
IDA: Durch innovative Testverfahren Darmkrebs früher erkennen (German name)
IQR: interquartile range
ROC: receiver operating characteristic
sCEA: serum carcinoembryonic antigen
SD: standard deviation
SOPs: standard operating procedures

## 1 INTRODUCTION

Colorectal cancer (CRC) is one of the leading causes of cancer‐related deaths worldwide, with an estimated 1.9 million new cases and over 0.9 million deaths in 2022 alone.^1^ The high mortality rate is primarily due to late‐stage diagnosis, highlighting the urgent need for effective early diagnosis and detection methods.

Currently, several methods are used for CRC detection or screening, including colonoscopy, flexible sigmoidoscopy, fecal occult blood testing (FOBT) performed mostly by fecal immunochemical test (FIT).^2^, ^3^ Colonoscopy is widely regarded as the gold standard for detecting CRC and precancerous lesions.^4^, ^5^ Despite its clinical effectiveness, its widespread adoption for CRC screening is limited due to invasiveness, the need for bowel preparation, and high cost.^6^, ^7^ Flexible sigmoidoscopy is less invasive and costly than colonoscopy and does not require complete bowel preparation or sedation. However, it misses most neoplasms located in the proximal colon.^8^, ^9^ FIT is a more convenient and inexpensive option compared to colonoscopy; however, it has limited sensitivity for early‐stage CRC and its precursors.^10^, ^11^, ^12^ Therefore, there is a critical need for cost‐effective, non‐invasive, and easily applicable CRC detection tests with enhanced sensitivity and specificity.

Serum carcinoembryonic antigen (sCEA) has been one of the most extensively used non‐invasive blood biomarkers for CRC detection or monitoring for many years.^13^, ^14^, ^15^, ^16^ Although its expression is often elevated, particularly in advanced stages of the disease, its low sensitivity for detecting early‐stage tumors limits its utility in CRC screening.^13^ To improve diagnostic performance, several studies have evaluated sCEA in combination with other blood biomarkers. For example, Kildusiene et al.^17^ reported that combining sCEA with serum CA72‐4 and CA19‐9 obviously increased sensitivity for CRC detection compared to sCEA alone. Similarly, Wild et al.^18^ demonstrated that combining sCEA with CYFRA 21‐1, ferritin, osteopontin, anti‐p53, and seprase also significantly enhanced sensitivity than sCEA alone.

In parallel, CEA can be detected not only in serum but also in feces.^19^ Feces is a rich source of cells derived from the gastrointestinal tract that can be used to measure tumor‐related proteins such as CEA. Furthermore, the concentration of fecal CEA (fCEA) is higher than that of sCEA, especially in the early‐stage of CRC,^20^ which prompted researchers to advocate the use of fCEA for CRC detection. Several studies have suggested that fCEA might be a potential non‐invasive biomarker and more sensitive than sCEA for CRC detection.^19^, ^20^, ^21^

We recently conducted a systematic review to summarize the potential of fCEA for CRC detection.^22^ All seven included studies showed that fCEA concentrations were significantly higher in CRC cases than in controls. However, the diagnostic performance of fCEA in a non‐Asian population was evaluated in only one small study in the United Kingdom conducted in 1986, which did not include a comparison with the diagnostic performance of sCEA.^23^ Therefore, in this study, we performed a head‐to‐head comparison of the diagnostic value of fecal and serum CEA for CRC in study participants selected from two ongoing large CRC detection studies in Germany, aiming to determine whether fCEA yields superior diagnostic performance to sCEA for CRC detection and whether combining either marker with FIT improves performance.

## 2 MATERIALS AND METHODS

### 2.1 Study population

Patients with newly diagnosed CRC and controls free of colorectal neoplasm at screening colonoscopy were randomly selected from the IDA study and BLITZ study, respectively. In the IDA study (German name: Durch **i**nnovative Testverfahren **Da**rmkrebs früher erkennen), which is conducted in collaboration with a network of clinics in southern Germany, pre‐treatment blood and fecal samples are collected from patients with newly diagnosed CRC with the primary aim of evaluating the diagnostic performance of novel non‐invasive tests for CRC detection at various stages. In the BLITZ study (German name: **B**egleitende Eva**l**uierung **i**nnovativer **T**estverfahren **z**ur Darmkrebsfrüherkennung), which is carried out in collaboration with a network of gastroenterology practices in southern Germany, pre‐colonoscopy blood and fecal samples are collected from participants of screening colonoscopy offered as a primary screening examination in Germany since 2002. Details of the study designs of the IDA and BLITZ study have been reported elsewhere.^11^, ^24^

Participants for this analysis were recruited between 2008 and 2020 for the BLITZ study, and between 2013 and 2016 for the IDA study. Participants were excluded if any of the following criteria were met: (1) no serum or fecal samples were available; (2) serum and fecal samples were collected after colonoscopy or the blood collection date was unknown; (3) a history of CRC or inflammatory bowel disease; (4) colonoscopy within the past 5 years; and (5) inadequate bowel preparation or incomplete colonoscopy. Eligible participants were randomly selected from the study cohort using a computer‐generated random sequence in R (version 4.2.3), with a fixed random seed to ensure reproducibility. No additional matching on age, sex, or other factors was performed.

### 2.2 Sample processing

In both the IDA and the BLITZ studies, whole blood samples were collected and transported to the German Cancer Research Center (DKFZ) biobank under cold chain conditions after being drawn. The whole blood samples were first clotted at room temperature for 30 min and then centrifuged at 2000–2500 × g for 10 min at 4°C. The resulting supernatant (serum) was carefully collected and stored at −80°C until further processing. Fecal samples were collected in Sysmex ‘FOB Gold Tube Screen’ collecting tubes. Following the manufacturer's instructions, fecal collection sticks containing 10 mg of feces were inserted into 1.7 mL of preservation buffer. The fecal collecting tubes were vortexed evenly, and the contents were pipetted into six aliquots. Given that there is no established protocol for sample processing for fCEA measurements, we applied and compared two different methods to process the fecal samples. With the first sample processing method, one of the six aliquots was centrifuged at 20,000 g for 15 min, and the supernatant was diluted in a 1:10 ratio with the preservation buffer for fCEA analyses. With the second sample processing method, another aliquot was transferred into a new tube with ceramic beads (diameter = 1.4 mm, *n* = 20), and then ground using a Precellys24 Homogenizer at 5200 g for 45 s at 4°C, followed by centrifugation at 18,000 g for 15 min at 4°C. Finally, the supernatant was extracted and diluted 1:100 with the preservation buffer. The first sample processing method (without homogenization) was chosen to represent a commonly used, minimally processed approach in stool‐based biomarker analysis. The rationale for the second sample processing method (with homogenization) was that fCEA may be present mainly in cells, so that adequate mechanical homogenization of the fecal sample is required to break the cells in the feces and release the intracellular CEA. Comparing both approaches allowed us to assess whether the additional homogenization step provides meaningful analytical or diagnostic advantages before considering its implementation in future practice.

### 2.3 Sample measurements

The fCEA and sCEA concentrations were measured from samples collected during the same period and processed in parallel. For all study participants, 400 μL of diluted fecal supernatant and 400 μL of serum—both obtained from the same individuals—were used for the respective assays. Both fCEA and sCEA assays were performed simultaneously under identical technical conditions in the central lab of the University Hospital Heidelberg using an ‘ADVIA Centaur XPT Immunoassay‐System (Siemens Healthineers) according to standard operating procedures (SOPs), including routine internal calibration and quality controls. While specific inter‐assay coefficients of variation were not calculated for this study, the laboratory follows strict SOPs to ensure reliable and reproducible results. Laboratory personnel performing the assays were blinded to case–control status to minimize bias. In addition, all participants underwent FIT value testing using the FIT test from Sentinel Diagnostics. FIT analyses were performed at the Limbach Laboratory (Heidelberg, Germany) on the Abbott Architect c8000. Participants were categorized as FIT positive or negative according to the manufacturer's recommended cutoff value (100 ng hemoglobin per mL buffer/17 μg hemoglobin per gram feces).

### 2.4 Statistical analyses

All data were analyzed using R version 4.2.3 and MedCalc Statistical Software version 22.019. Categorical variables were reported as numbers (percentages), and continuous variables were presented as medians [interquartile ranges, IQR]. Correlations between fCEA concentrations and sCEA concentrations were assessed by Spearman rank correlation coefficients. Categorical variables were compared between the CRC group and the control group using the χ2 test. Continuous variables were compared using the Mann–Whitney U test. Differences among three groups were evaluated using one‐way ANOVA, with Dunn's multiple comparisons test applied following a significant Kruskal–Wallis test. The diagnostic performance of fCEA and sCEA levels for detecting CRC was evaluated by calculating the area under the receiver operating characteristic curve (AUC) with 95% confidence interval (CI) and sensitivities with 95% CI at cut‐off values yielding a predefined and fixed specificity of 85%. For the AUC analysis, continuous variables such as sCEA and fCEA were analyzed without predefined cut‐off values, while FIT values were dichotomized based on the manufacturer's recommended cutoff. Comparisons between AUCs were performed using DeLong's test. Sensitivities were compared using McNemar's test. We furthermore carried out forest plots and receiver operating characteristic curve (ROC) analyses to evaluate if and to what extent the diagnostic performance of FIT could be improved by the combination of FIT results with fCEA or sCEA results. As FIT is widely used as a dichotomous test with a fixed cutoff and is approved only as a dichotomous test by the Food and Drug Administration in the United States, our analyses were carried out using either the dichotomous FIT result (using the cutoff recommended by the manufacturer) or the quantitative FIT result. Statistical significance was defined by two‐sided *p*‐value <.05.

## 3 RESULTS

### 3.1 Characteristics of the study participants

**FIGURE 1:**
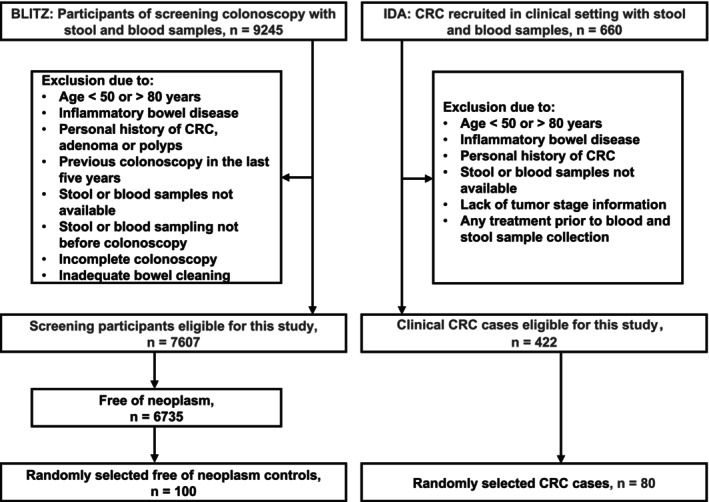
Study flow diagram of participants. BLITZ, Begleitende Evaluierung innovativer Testverfahren zur Darmkrebsfrüherkennung (German) study; CRC, colorectal cancer; IDA, Durch innovative Testverfahren Darmkrebs früher erkennen (German) study.

Figure 1 provides a study flow diagram illustrating the selection of study participants from the BLITZ study (*n* = 9245) and the IDA study (*n* = 660). A total of 80 CRC cases from the IDA study and 100 controls free of colorectal neoplasms from the BLITZ study were randomly selected from the eligible participants. Demographical and clinical characteristics of participants in the CRC group and in the control group are shown in Table 1. The mean age was 62.6 ± 7.7 years in the CRC group and 59.7 ± 7.1 years in the control group, with median ages of 62 years (IQR: 56–70) and 58 years (IQR: 55–64), respectively. Females accounted for 45.0% of CRC cases and 60.0% of controls. No participants in either group were underweight. In the CRC group, 31.2% were overweight and 32.5% were obese, compared with 42.0% and 22.0% in the control group. The CRC group consisted of patients with colon cancer (56.3%) and rectal cancer (43.7%). Among them, 50% were diagnosed with early‐stage CRC (stages 0, I, and II).

**TABLE 1:** Characteristics of the study participants.

Characteristic	CRC cases *n* = 80	Controls *n* = 100
Age, year		
Mean (SD)	62.6 (7.7)	59.7 (7.1)
Median [IQR]	62 [56–70]	58 [55–64]
Sex, *n* (%)		
Male	44 (55.0)	40 (40.0)
Female	36 (45.0)	60 (60.0)
BMI, kg/m^2^, *n* (%)		
Underweight (<18.5)	0 (0)	0 (0)
Normal (18.5–24.9)	29 (36.3)	36 (36.0)
Overweight (25–29.9)	25 (31.2)	42 (42.0)
Obese (≥30)	26 (32.5)	22 (22.0)
Tumor site, *n* (%)		
Colon	45 (56.3)	—
Rectum	35 (43.7)	—
Tumor stage, *n* (%)		
0	3 (3.8)	
I	25 (31.2)	—
II	12 (15.0)	—
III	25 (31.2)	—
IV	15 (18.8)	—

*Note*: Categorical variables are reported as numbers (%), and age distributions are presented as medians [interquartile ranges, IQR]. Tumor stages were determined by the Union for International Cancer Control (UICC) classification (8th edition).

Abbreviations: BMI, body mass index; CRC, colorectal cancer; IQR, interquartile range; *n*, number; SD, standard deviation.

### 3.2 Correlation between fCEA and sCEA concentrations, and comparison of them between the CRC group and the control group

The fCEA concentration was not significantly correlated with sCEA concentration, regardless of the fecal sample processing method or participant group. However, fCEA concentrations obtained by the two sample processing methods were almost perfectly correlated in both the CRC group (r = 0.96, *p* < .001) and the control group (r = 0.95, *p* < .001) (Table S1). The median sCEA concentration was significantly higher in the CRC group than in the control group (3.2 [1.7–8.4] vs. 0.5 [0.2–2.4] μg/L, *p* < .001). In contrast, fCEA did not show consistent differences between CRC cases and controls across the two fecal processing methods; a significant difference was observed only with the first processing method (176.8 [83.1–279.7] vs. 126.1 [67.1–184.4] ng/mg, *p* = .006), but not with the second method (177.1 [81.2–257.8] vs. 143.8 [72.1–203.1] ng/mg, *p* = .125) (Table 2).

**TABLE 2:** Concentrations of fCEA and sCEA among CRC cases and controls.

Groups (*n*)	First sample processing method		Second sample processing method			
	fCEA (ng/mg)	*p*	fCEA (ng/mg)	*p*	sCEA (μg/L)	*p*
CRC (80)	176.8 [83.1–279.7]	.006*	177.1 [81.2–257.8]	.125	3.2 [1.7–8.4]	<.001*
Control (100)	126.1 [67.1–184.4]	ref	143.8 [72.1–203.1]	ref	0.5 [0.2–2.4]	ref

*Note*: Continuous variables are presented as medians [interquartile ranges].

Abbreviations: CRC, colorectal cancer; fCEA, fecal carcinoembryonic antigen; *n*, number; ref., reference; sCEA, serum carcinoembryonic antigen.

* *p* < .05.

### 3.3 Subgroup analysis stratified by the clinical characteristics of the CRC group

In subgroup analyses of CRC cases, fCEA concentrations were examined according to clinical characteristics including age, sex, tumor site, and tumor stage. The fCEA concentrations varied significantly by tumor site and tumor stage across both fecal sample processing methods. Both median fCEA concentrations were significantly higher in colon cancer patients (*n* = 45) than in rectal cancer patients (*n* = 35): 228.9 (101.3–318.1) vs. 146.2 (65.7–202.3), *p* = .010; 206.4 vs. 133.6 ng/mg, *p* = .009. Similarly, both of the median fCEA concentrations were significantly higher in late‐stage CRC (stage III and IV, *n* = 40) compared to early‐stage CRC (stage 0, I, and II, n = 40): 230.9 (97.9–341.5) vs. 134.6 (64.9–228.5) ng/mg, *p* = .021; 199.4 (98.6–283.5) vs. 142.5 (68.0–217.7) ng/mg, *p* = .047. The sCEA concentrations showed a significant difference only by tumor stage, with late‐stage CRC exhibiting significantly higher median concentrations than early‐stage CRC (4.8 [2.0–43.3] vs. 2.3 [1.6–5.5] ng/mg, *p* = .013) (Table 3).

**TABLE 3:** Subgroup analysis based on clinical characteristics in the CRC group.

Characteristic (*n*)	First sample processing method		Second sample processing method			
	fCEA (ng/mg)	*p*	fCEA (ng/mg)	*p*	sCEA (μg/L)	*p*
Age		.368		.226		.077
<65 (45)	177.8 [94.9–308.0]		180.9 [93.9–273.7]		2.4 [1.5–6.5]	
≥65 (35)	175.8 [66.6–248.4]		163.2 [68.0–220.0]		4.8 [2.0–9.6]	
Sex		.382		.592		.089
Male (44)	181.7 [105.1–268.3]		178.5 [82.5–252.9]		2.4 [1.4–8.0]	
Female (36)	165.9 [60.7–283.2]		172.4 [77.9–257.8]		4.8 [1.9–8.7]	
BMI		.153		.160		.524
Normal (29)	134.6 [66.2–243.2]	ref	151.3 [70.2–220.3]	ref		ref
Overweight (25)	198.4 [95.4–250.6]	.104	182.1 [89.7–219.7]	.103		.963
Obese (26)	187.7 [89.59–376.4]	.388	173.7 [83.8–302.3]	.595		.448
Tumor site		.010*		.009*		.438
Colon (45)	228.9 [101.3–318.1]		206.4 [102.9–289.9]		3.6 [1.7–9.2]	
Rectum (35)	146.2 [65.7–202.3]		133.6 [66.3–200.3]		2.4 [1.8–6.0]	
Tumor stage		.021*		.047*		.013*
Early‐stage (0 + I + II, 40)	134.6 [64.9–228.5]		142.5 [68.0–217.7]		2.3 [1.6–5.5]	
Late‐stage (III + IV, 40)	230.9 [97.9–341.5]		199.4 [98.6–283.5]		4.8 [2.0–43.3]	

*Note*: Continuous variables are presented as medians [interquartile ranges].

Abbreviations: BMI, body mass index; CRC, colorectal cancer; fCEA, fecal carcinoembryonic antigen; *n*, number; ref., reference; sCEA, serum carcinoembryonic antigen.

* *p* < .05.

### 3.4 Diagnostic performance of fCEA and sCEA for CRC detection

**FIGURE 2:**
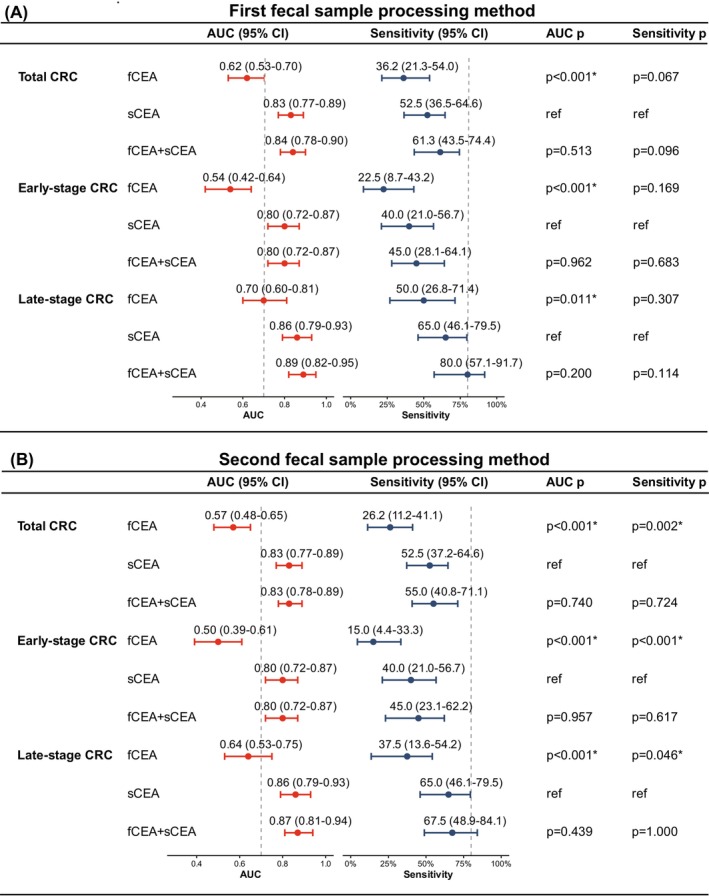
Diagnostic performance of fCEA, sCEA, and their combination. Forest plots show the AUC with 95% CIs (red panels) and sensitivity with 95% CIs at a fixed specificity of 85% (blue panels) for fCEA, sCEA, and their combination (fCEA + sCEA) in the detection of total CRC, early‐stage CRC (stages 0, I, and II), and late‐stage CRC (stages III and IV). (A): First fecal sample processing method. (B): Second fecal sample processing method. AUC, area under the curve; CI, confidence interval; CRC, colorectal cancer; fCEA, fecal carcinoembryonic antigen; ref., reference; sCEA, serum carcinoembryonic antigen. *p < .05.

As shown in Figure 2A and Figure S1, using the first fecal sample processing method, fCEA alone showed limited discriminatory ability for the detection of total CRC, with an AUC (95% CI) of 0.62 (0.53–0.70), whereas sCEA demonstrated substantially higher diagnostic performance, with an AUC of 0.83 (0.77–0.89). Combining fCEA with sCEA resulted in an AUC of 0.84 (0.78–0.90), which was not significantly different from that of sCEA alone (*p* = .513). At a predefined specificity of 85%, fCEA exhibited low sensitivity for total CRC detection (36.2%), whereas sCEA achieved a sensitivity exceeding 50%, and the combined model did not materially improve sensitivity compared with sCEA alone (61.3% vs. 52.5%, *p* = .096). For early‐stage CRC (stages 0–II), fCEA showed poor diagnostic performance, with an AUC of 0.54 (0.42–0.64). In contrast, both sCEA and the combined model achieved moderate discrimination, each with an AUC of 0.80 (0.72–0.87). The addition of fCEA to sCEA did not result in a significant improvement in AUC (*p* = .962) or sensitivity (*p* = .683), indicating no incremental value of fCEA for early‐stage CRC detection. In contrast, for late‐stage CRC (stages III–IV), sCEA alone demonstrated high diagnostic accuracy, with an AUC of 0.86 (0.79–0.93). The combined model yielded a higher AUC of 0.89 (0.82–0.95); however, this increase was not statistically significant compared with sCEA alone (*p* = .200). Although the combined model showed the highest sensitivity (80.0% vs. 65.0%), the difference in sensitivity relative to sCEA alone was not statistically significant (*p* = .114).

Consistent patterns were observed using the second fecal sample processing method (Figure 2B). Across total CRC, early‐stage CRC, and late‐stage CRC, fCEA alone consistently showed lower AUCs (0.57, 0.50, and 0.64, respectively) and lower sensitivities at 85% specificity (26.2%, 15.0%, and 37.5%, respectively), whereas sCEA maintained superior diagnostic performance, with AUCs of 0.83, 0.80, and 0.86 and corresponding sensitivities of 52.5%, 40.0%, and 65.0%. The combined model did not significantly outperform sCEA alone in terms of either AUC or sensitivity in any CRC subgroup. These findings indicate that sCEA is the primary contributor to diagnostic performance in both fecal sample processing methods, and that combining fCEA with sCEA does not materially increase diagnostic performance, particularly for the detection of early‐stage CRC.

### 3.5 Diagnostic performance of FIT alone, FIT combined with fCEA, and FIT combined with sCEA for CRC detection

**FIGURE 3:**
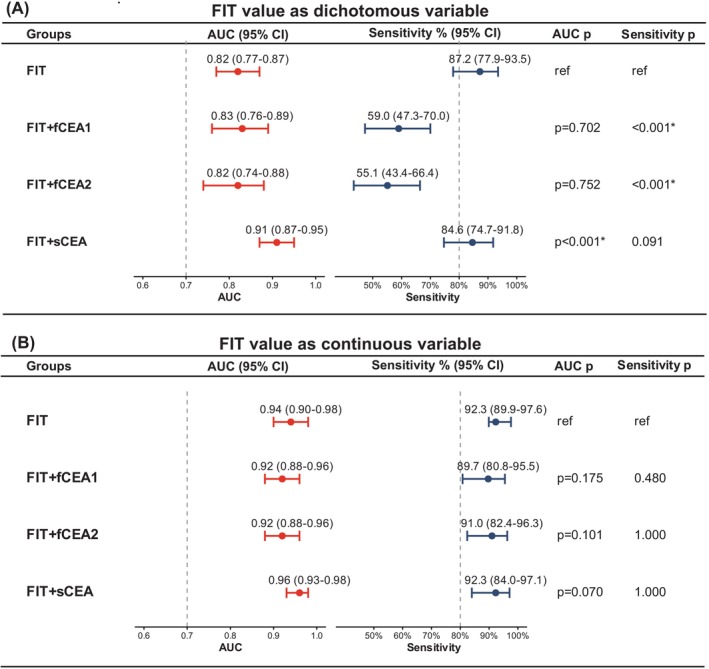
Diagnostic performance of FIT alone, FIT combined with fCEA, and FIT combined with sCEA. Forest plots illustrate the AUC with 95% CIs (red panels) and sensitivity with 95% CIs at a fixed specificity of 85% (blue panels) for FIT alone and in combination with fCEA1 (first fecal sample processing method) or fCEA2 (second fecal sample processing method) or sCEA, evaluated using FIT values as a dichotomous variable according to the manufacturer's recommended cutoff value (A) and as a continuous variable (B). AUC, area under the curve; CI, confidence interval; FIT, fecal immunochemical test; fCEA, fecal carcinoembryonic antigen; ref., reference; sCEA, serum carcinoembryonic antigen. *p < .05.

As shown in Figure 3A and Figure S2, when FIT was treated as a dichotomous variable using the manufacturer‐recommended cutoff, FIT alone demonstrated good discriminatory ability for colorectal cancer detection, with an AUC (95% CI) of 0.82 (0.77–0.87). The addition of fCEA (from either fecal sample processing method) to FIT did not significantly improve AUCs of 0.83 (0.76–0.89) and 0.82 (9 0.74–0.88), respectively (*p* = .702 and *p* = .752 vs. FIT). In contrast, combining FIT with sCEA resulted in a significantly higher AUC of 0.91 (0.87–0.95) compared with FIT alone (*p* < .001). At a fixed specificity of 85%, FIT alone achieved a sensitivity of 87.2% (77.9–93.5). The addition of fCEA to FIT was associated with substantially lower sensitivities (*p* < .001).

When FIT was analyzed as a continuous variable, overall diagnostic performance further improved. FIT alone achieved an AUC of 0.94 (0.90–0.98). The addition of fCEA to FIT did not increase discrimination [AUCs of 0.92 (0.88–0.96) for both fecal sample processing methods; *p* = .175 and *p* = .101, respectively]. Combining FIT with sCEA yielded the highest AUC of 0.96 (95% CI, 0.93–0.98). Sensitivity at 85% specificity remained high across all continuous FIT–based models, exceeding 89%, with no significant differences observed following the addition of either fCEA or sCEA (all *p* ≥ .480) (Figure 3B).

## 4 DISCUSSION

Although sCEA has long been widely used as one of the biomarkers for the diagnosis of CRC in clinical practice and screening cohorts,^25^, ^26^ evidence identifying the role of fCEA in CRC detection has been very limited. Fecal samples are easily accessible, and fCEA testing is appealing from a practical standpoint; however, our results suggest that fCEA concentrations in the CRC group showed a less distinct difference from the control group compared to sCEA concentrations. Accordingly, the diagnostic performance (AUC value and sensitivity) of fCEA was much lower than that of sCEA, which suggests that fCEA may not be a more promising biomarker for CRC diagnosis compared with sCEA.

Given that a single laboratory test biomarker is difficult to serve as a definitive diagnostic or screening tool for CRC,^25^, ^27^ we also explored the potential to enhance diagnostic performance by combining fCEA and sCEA with FIT, the most widely‐used non‐invasive test for CRC screening.^28^, ^29^ We found that the diagnostic performance of FIT combined with fCEA was lower than that of FIT combined with sCEA, indicating that FIT combined with fCEA provides less potential for CRC detection compared with FIT combined with sCEA. Therefore, replacing sCEA with fCEA is not supported. Rather, our findings suggest that sCEA should be prioritised, particularly when combined with FIT, for non‐invasive CRC detection.

Notably, fCEA concentrations were significantly higher in colon cancer patients than in rectal cancer patients for both sample processing methods. This could be attributed to the following two reasons: First, the environmental conditions within the colon might favor the accumulation of CEA more than those within the rectum. For example, the transit time of fecal matter through the colon is longer,^30^ which might allow more CEA to be secreted and retained, resulting in higher detectable fCEA concentrations in colon cancer patients. Second, colon cancers are often diagnosed at a later stage than rectal cancers,^31^ potentially leading to larger tumor sizes and more CEA production and secretion to feces. Additionally, we found that fCEA and sCEA levels were significantly lower in early‐stage CRC compared to late‐stage CRC. Tomašević et al.^32^ also reported that sCEA levels in early‐stage CRC were significantly lower than those in late‐stage CRC. However, to our knowledge, no other studies have reported significantly different fCEA levels in early‐ and late‐stage CRC.

Until now, few studies have directly compared the diagnostic value of fCEA and sCEA for CRC detection, making our study a valuable contribution to the literature. However, our results are inconsistent with two recent studies from China with overlapping study populations, which reported significantly higher fCEA concentrations in CRC cases than in non‐gastrointestinal cancer cases and healthy controls, and a higher AUC value for fCEA than for sCEA concentrations (0.802 vs. 0.757) in distinguishing CRC cases from controls.^20^, ^21^ Another study from South Korea by Kim, et al.^19^ reported a higher sensitivity (85.70% vs. 39.29%), but a lower specificity (92.96% vs. 96.66%) of fCEA than of sCEA for CRC detection. One possible reason for the discrepancy could be differences in ethnic and population characteristics. Our study predominantly included white participants from Germany, whereas the other studies mentioned above were conducted in East Asian countries. Another reason could be variations in fecal sample processing methods and diagnostic criteria. The methodologies used to process fecal samples and measure fCEA and sCEA concentrations can differ significantly between studies. Differences in assay sensitivity, specificity, and cut‐off values for defining positive results could lead to varying diagnostic accuracy. It is crucial to standardize these methodologies to ensure comparability across studies. In addition, another reason for the apparent discrepancy between the South Korean study and our study could be publication bias. It appears plausible that the seemingly quite promising results reported in the South Korean study published in 2003 would have been taken up and replicated by other researchers. The absence of reported replication of such promising results over 20 years suggests that unsuccessful attempts at such replication may have gone unpublished due to lack of significant findings.

In the interpretation of our study, several limitations require careful consideration. Firstly, while our study provides initial insights contrasting diagnostic values of fCEA and sCEA for CRC, the small sample size is a limitation. Further research with larger study populations is essential to confirm our findings. However, in contrast to fCEA, sCEA showed statistically highly significant differences with more than 6‐fold higher mean concentrations in the CRC group than in the control group. Furthermore, differences between the diagnostic performance of fCEA and sCEA were statistically significant despite the limited sample size. Secondly, there is currently no standardized method for processing fecal samples for fCEA detection. Even though we employed two fecal sample processing methods that showed near‐perfect correlation, we acknowledge that fCEA measurements may still be more susceptible to technical variability than sCEA due to the complex stool matrix and potential differences in sample handling. Moreover, unknown differences may exist compared with previously published studies, and the reproducibility of these methods across different laboratories remains unclear. Future research should focus on harmonizing study protocols, including standardized sample collection, processing, measurement, and analysis methods. Finally, participants were drawn from two ongoing CRC detection studies conducted in different time periods (IDA, 2013–2016; BLITZ, 2008–2020), which may introduce potential selection bias, as differences in eligibility criteria, participant characteristics, clinical practices, or sample handling between the two study periods could affect the comparability of the groups. Collaborative efforts across multiple centers and countries, including larger numbers of participants from true screening settings, may provide more robust data and help clarify the detection value of fCEA and sCEA for CRC.

## 5 CONCLUSIONS

Our head‐to‐head comparison does not support suggestions that fCEA may be a more promising non‐invasive biomarker than sCEA for CRC detection. The combination of FIT with sCEA demonstrates greater potential for CRC detection than the combination of FIT with fCEA. Our findings offer investigators a new perspective of evidence on the role of fCEA in CRC detection and call for well‐designed, large‐scale prospective studies that would clarify existing controversies.

## AUTHOR CONTRIBUTIONS

**Xianzhe Li:** Conceptualization; methodology; software; investigation; formal analysis; data curation; visualization; writing – original draft; writing – review and editing. **Zitong Zhao:** Methodology; software; formal analysis; investigation; writing – review and editing. **Lara Stassen:** Methodology; validation; writing – review and editing. **Anjana Pradeep Maya:** Methodology; validation; writing – review and editing. **Megha Bhardwaj:** Writing – review and editing. **Teresa Seum:** Writing – review and editing. **Janhavi R. Raut:** Writing – review and editing. **Tafirenyika Gwenzi:** Writing – review and editing. **Michael Hoffmeister:** Resources; writing – review and editing. **Petra Schrotz‐King:** Methodology; resources; data curation; writing – review and editing; project administration; supervision. **Hermann Brenner:** Conceptualization; methodology; resources; writing – review and editing; supervision; funding acquisition; project administration.

## FUNDING INFORMATION

This work was supported in part by grants from the Federal Ministry of Education and Research (BAMF, grant no.: 01KD2104A).

## CONFLICT OF INTEREST STATEMENT

The authors declare no conflicts of interest.

## ETHICS STATEMENT

The study was conducted in accordance with the Declaration of Helsinki. The use of samples from the IDA study was approved by the ethics committees of the Medical Faculty Heidelberg (S‐489/2012). The use of samples from the BLITZ study was approved by the ethics committees of the Medical Faculty Heidelberg (S‐178/2005), and of the responsible state physicians' chambers (Baden‐Württemberg, M118‐05‐f; Rhineland‐Palatinate, 837.047.06[5145]; Saarland, 217/13; Hesse, MC 254/2007). Informed consent was obtained from all subjects involved in the study.

## Supporting information


**Data S1** Supporting Information.

## Data Availability

The original contributions presented in the study are included in the article; further inquiries can be directed to the corresponding author. Data may be made available upon reasonable request within the limitations imposed by the informed consent and legal requirements.
