# Supplementary material for: Head‐to‐head comparison of the diagnostic value of fecal and serum carcinoembryonic antigen for colorectal cancer detection
Source: Int J Cancer. 2026 Apr 9;159(2):516–25. doi: 10.1002/ijc.70435 (PMC13193527; doi:10.1002/ijc.70435)
Supplement: Supplementary file 1 — Data S1 Supporting Information. [file IJC-159-516-s001.pdf]

Head-to-head comparison of the diagnostic value of fecal and serum carcinoembryonic antigen for colorectal cancer detection

Authors: Xianzhe Li, Zitong Zhao, Lara Stassen, Anjana Pradeep Maya, Megha Bhardwaj, Teresa Seum, Janhavi R. Raut, Tafirenyika Gwenzi, Michael Hoffmeister, Petra Schrotz-King and Hermann Brenner

Table of Contents

1. Supplementary Tables

Supplementary Table 1. Correlation between fCEA and sCEA measurements.

2. Supplementary Figures

Supplementary Figure 1. Comparison of fCEA, sCEA, and their combination in receiver operating characteristic analysis.

Supplementary Figure 2. Comparison of FIT alone, FIT combined with fCEA, and FIT combined with sCEA in receiver operating characteristic analysis.

Supplementary Tables

Supplementary Table 1. Correlation between fCEA and sCEA measurements.

Variable	r value	p
**fCEA using the first sample processing method and sCEA[a]**		
CRC (IDA)	-0.03	0.759
Controls (BLITZ)	0.11	0.286
**fCEA using the second sample processing method and sCEA[b]**		
CRC (IDA)	0.01	0.957
Controls (BLITZ)	0.09	0.396
**fCEAs between the two methods[c]**		
CRC (IDA)	0.96	< 0.001*
Controls (BLITZ)	0.95	< 0.001*

Note: Spearman rank correlation analysis was performed. Abbreviations: CRC, colorectal cancer; fCEA, fecal carcinoembryonic antigen; n, number; sCEA, serum carcinoembryonic antigen. * $p < 0.05$

[a]Correlation analysis between fCEA concentration using the first sample processing method and that of sCEA.

[b]Correlation analysis between fCEA concentration using the second sample processing method and that of sCEA.

[c]Correlation analysis between fCEA concentrations using the first and the second sample processing.

Supplementary Figures

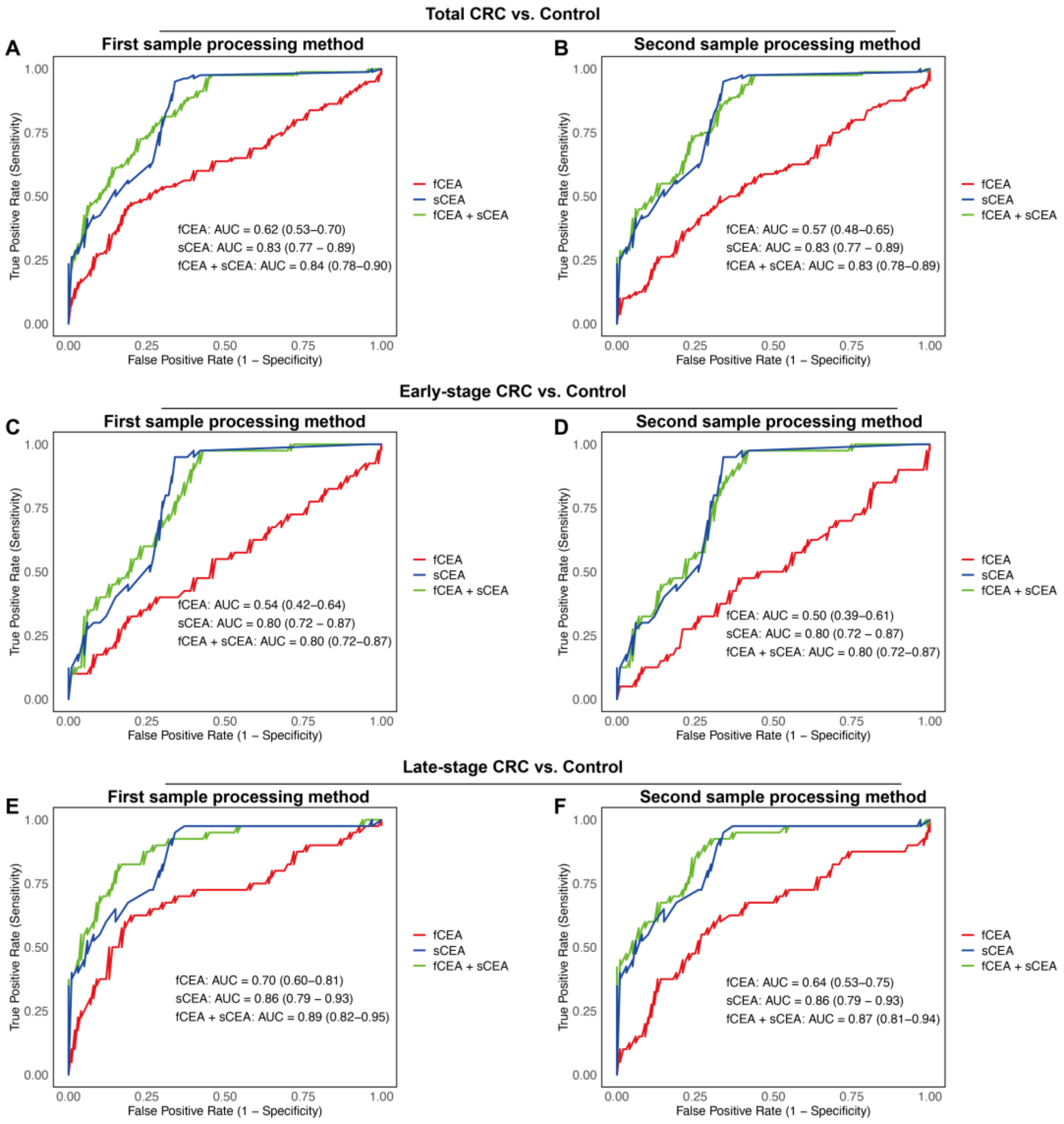

Supplementary Figure 1. Comparison of fCEA, sCEA, and their combination in receiver operating characteristic analysis. **A and B**: Comparison of fCEA, sCEA, and their combination between total CRC and control groups using two fecal sample processing methods. **C and D**: Comparison of fCEA, sCEA, and their combination between early-stage CRC and control groups using two fecal sample processing methods. **E and F**: Comparison of fCEA, sCEA, and their combination between late-stage CRC and control groups using two fecal sample processing methods. Abbreviations: AUC, Area under curve; CRC, colorectal cancer; fCEA, fecal carcinoembryonic antigen; sCEA, serum carcinoembryonic antigen.

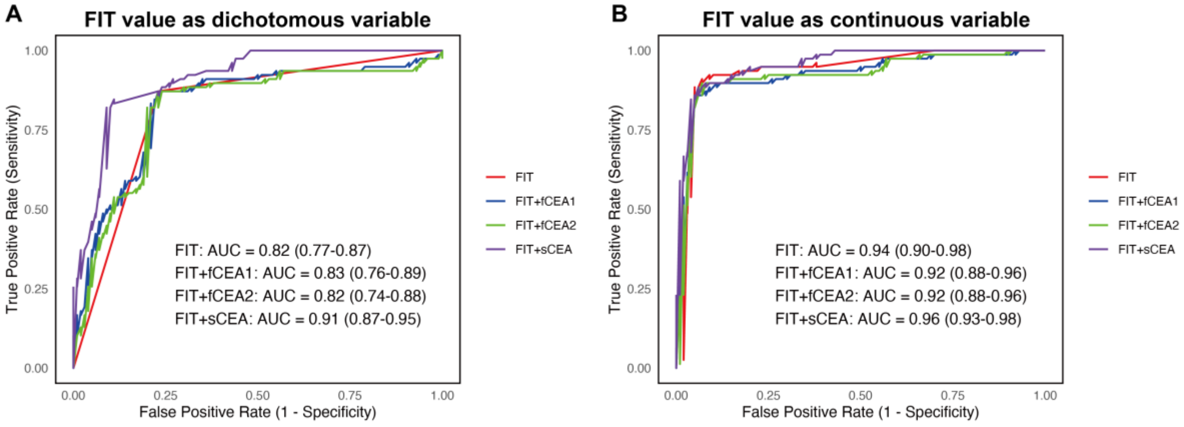

Supplementary Figure 2. Comparison of FIT alone, FIT combined with fCEA, and FIT combined with sCEA in receiver operating characteristic analysis. **A**: Comparison when the FIT value was used as a dichotomous variable based on the manufacturer's recommended cutoff. **B**: Comparison when FIT value was used as a continuous variable.

Abbreviations: AUC, Area under curve; CRC, colorectal cancer; FIT, fecal immunochemical test; fCEA1, fecal carcinoembryonic antigen value of the first fecal sample processing method; fCEA2, fecal carcinoembryonic antigen value of the second fecal sample processing method; sCEA, serum carcinoembryonic antigen.
